# Case report: Complete cast formation of the entire intrahepatic biliary tree

**DOI:** 10.3389/fmed.2025.1525442

**Published:** 2025-02-19

**Authors:** Pusen Wang, Lin Zhong, Dong Zhao

**Affiliations:** Department of Liver Surgery and Organ Transplantation Center, Shenzhen Third People’s Hospital, Second Affiliated Hospital, Southern University of Science and Technology, Shenzhen, Guangdong, China

**Keywords:** liver transplantation, biliary complication, non-anastomotic stricture, bile cast syndrome, biliary tree

## Abstract

Biliary complications pose substantial challenges in liver transplantation (LT), particularly with regard to non-anastomotic strictures (NASs). Among these ischemic-type biliary complications, bile cast syndrome (BCS) is a particularly severe condition. Currently, there are limited data on the formation of complete hepatic casts in BCS. In this case report, we present a unique instance of a second LT involving the formation of a complete hepatic bile cast and discuss its diagnostic and therapeutic process. This case aims to enhance the understanding of the anatomical and pathological features of BCS.

## Introduction

Liver transplantation (LT) is widely recognized as the optimal therapeutic approach for end-stage liver disease (1). Biliary complications, which are frequently observed post-transplantation, remain a significant source of morbidity in LT recipients. Biliary complications can be categorized into anastomotic stricture (AS) complications and non-anastomotic stricture (NAS) complications based on therapeutic aspects (2). The management of biliary complications poses considerable challenges, especially for NASs, often necessitating repeated interventions or even secondary LT (3). Bile cast syndrome (BCS) represents a rare occurrence of NASs, which manifests as blackened hardened material conforming to the shape of bile ducts (4). Although some studies have reported on the morphological aspects of BCS, comprehensive images that fully depict complete hepatic bile cast formation are still limited (5). In this study, we present a unique case involving complete hepatic bile cast formation and its diagnostic and therapeutic process with the aim of enhancing understanding of NASs and BCS from both anatomical and pathophysiological perspectives.

## Case description

A 32-year-old man was admitted to the liver transplantation (LT) department with a 2-day history of jaundice and abdominal pain. 7 months prior, he had undergone ABO-incompatible LT for hepatitis B virus (HBV)-related cirrhosis and acute-on-chronic liver failure, with maintenance immunosuppressive therapy consisting of tacrolimus and mycophenolic acid. The donor was donated after brain death and was HCV-positive. Prior to LT, the recipient underwent plasma exchange twice and received two doses of 300 mg rituximab, resulting in an ABO antibody titer below 1:64. On admission, the patient presented without fever and exhibited stable vital signs. Additionally, the patient had a medical history of hip arthroplasty due to femoral head necrosis, received entecavir treatment for HBV prophylaxis, and achieved HCV eradication.

Physical examination revealed jaundiced skin and sclera, along with mild tenderness in the upper right abdomen. Laboratory findings included white blood cells 2.85 × 10^9^/L (reference 3.5–9.5 × 10^9^/L) with hemoglobin 119 g/L (reference 130–175 g/L), platelets 85 × 10^9^/L (reference 125–350 × 10^9^/L), C-reactive protein 4.62 mg/L (reference 0–6 mg/L), and procalcitonin 5.29 ng/mL (reference <0.1 ng/mL). Liver function tests revealed total bilirubin 262.8 μmol/L (reference 0–26 μmol/L), with albumin 34.8 g/L (reference 40–55 g/L), and aspartate aminotransferase 141 U/L (reference 0–45 U/L). Tacrolimus concentration was 22.2 ng/mL (reference 5–15 ng/mL). Hepatitis B serologic test revealed HBsAg 0.20 IU/mL (reference 0–0.05 IU/mL) with HBcAb 7.85 (reference <1). Kidney function revealed creatinine 134 μmol/L (reference 57–97) with urea 9.73 mmol/L (reference 3.1–8 mmol/L). Clotting function, thyroid function, gastrointestinal tumor markers, Epstein–Barr virus, cytomegalovirus, and coronavirus were all within normal limits.

**Figure 1:**
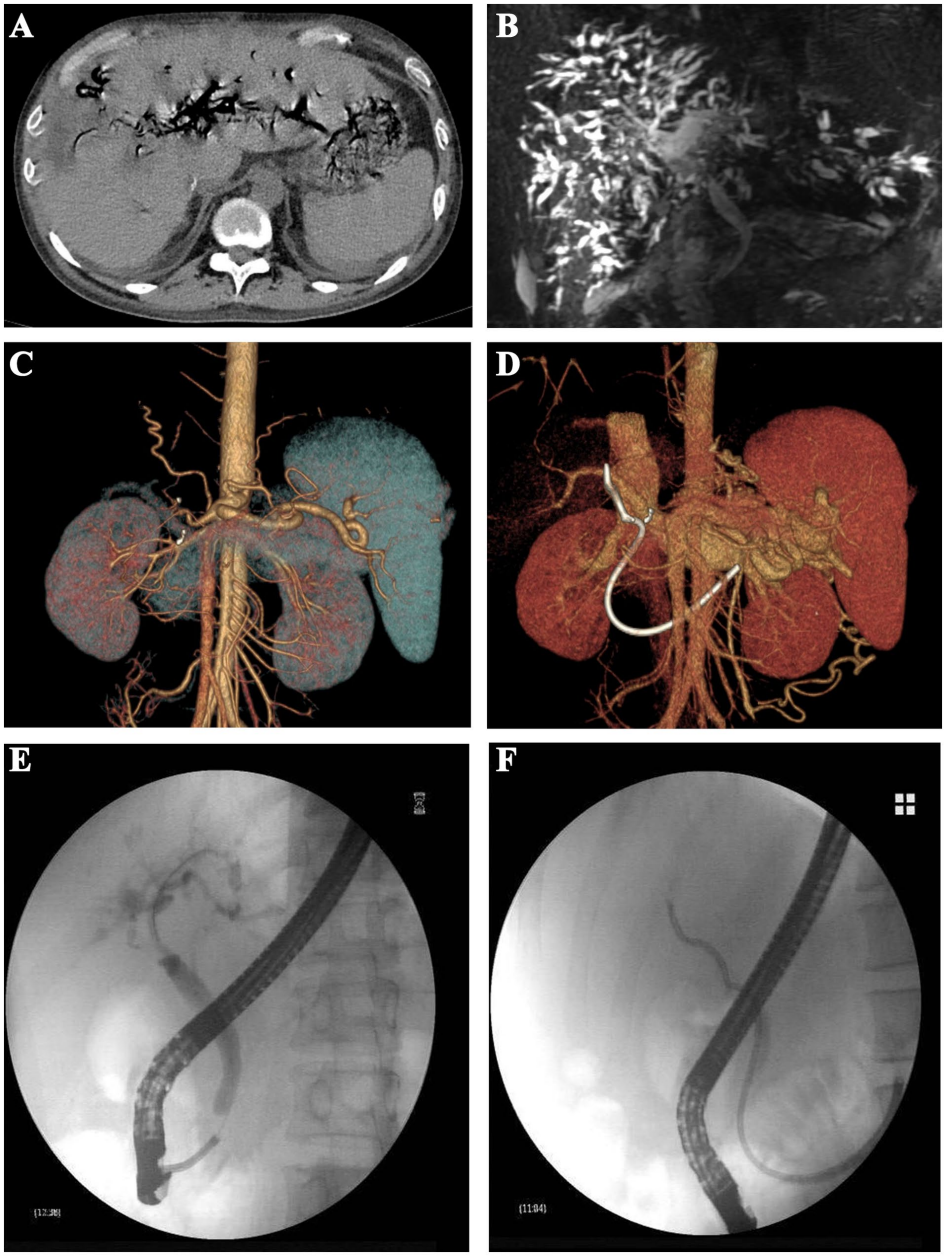
Imaging examinations and ERC procedures of the patient. **(A)** Abdominal contrast-enhanced computed tomography; **(B)** abdominal magnetic resonance cholangiopancreatography; **(C)** three-dimensional reconstruction of the hepatic artery; **(D)** three-dimensional reconstruction of the portal vein; **(E)** placement of biliary stents using ERC procedure; and **(F)** nasobiliary drainage using ERC procedure.

Multiple imaging tests, including abdominal contrast-enhanced computed tomography (CT) (Figure 1A) and magnetic resonance cholangiopancreatography (MRCP), demonstrated intrahepatic biliary ductal dilatation and stricture (Figure 1B). Abdominal CT angiography with three-dimensional reconstruction of the hepatic artery (Figure 1C) and portal vein (Figure 1D) revealed no evidence of anastomotic stenosis.

**Figure 2:**
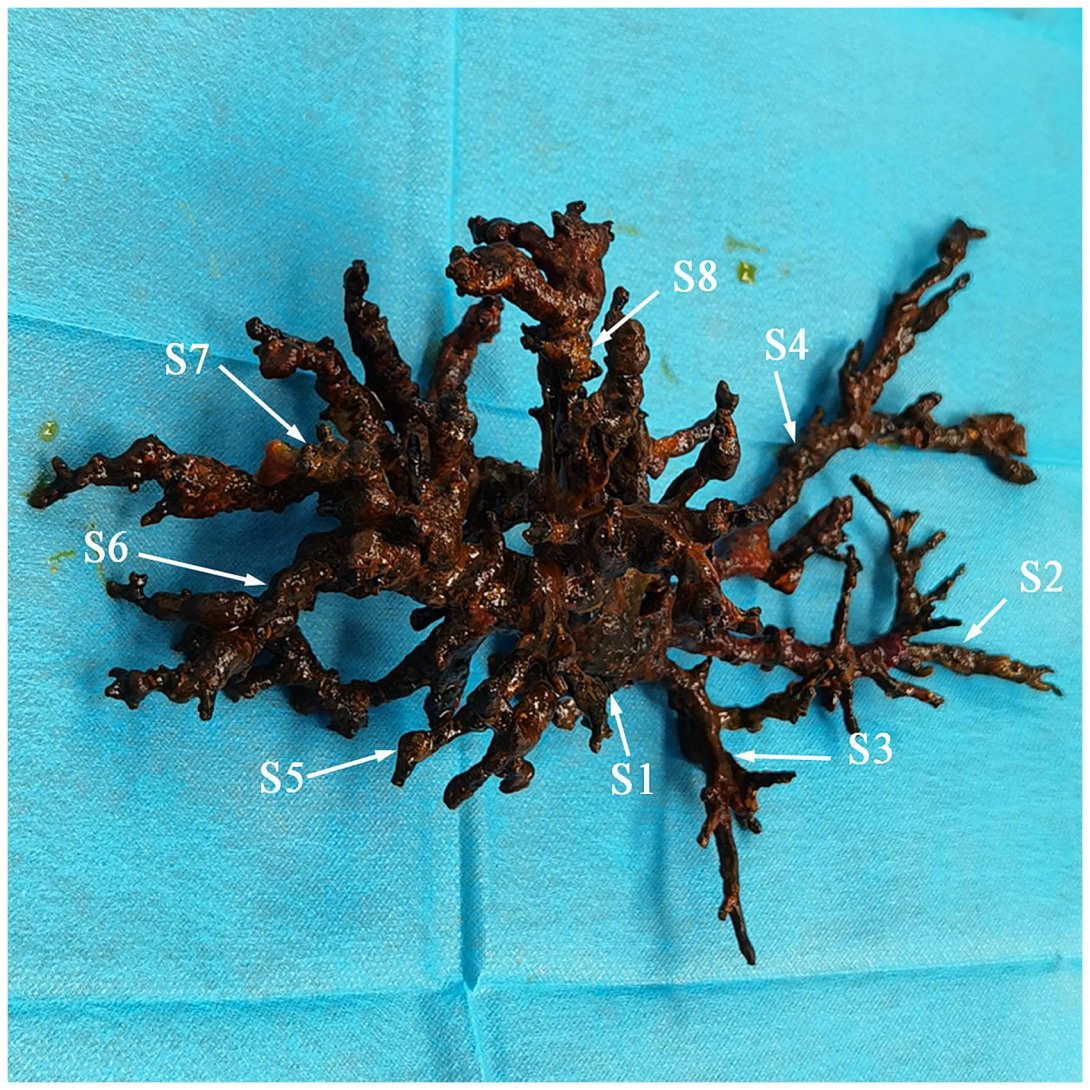
Complete casts within the entire biliary tract removed from the explanted liver (S, segment).

**Figure 3:**
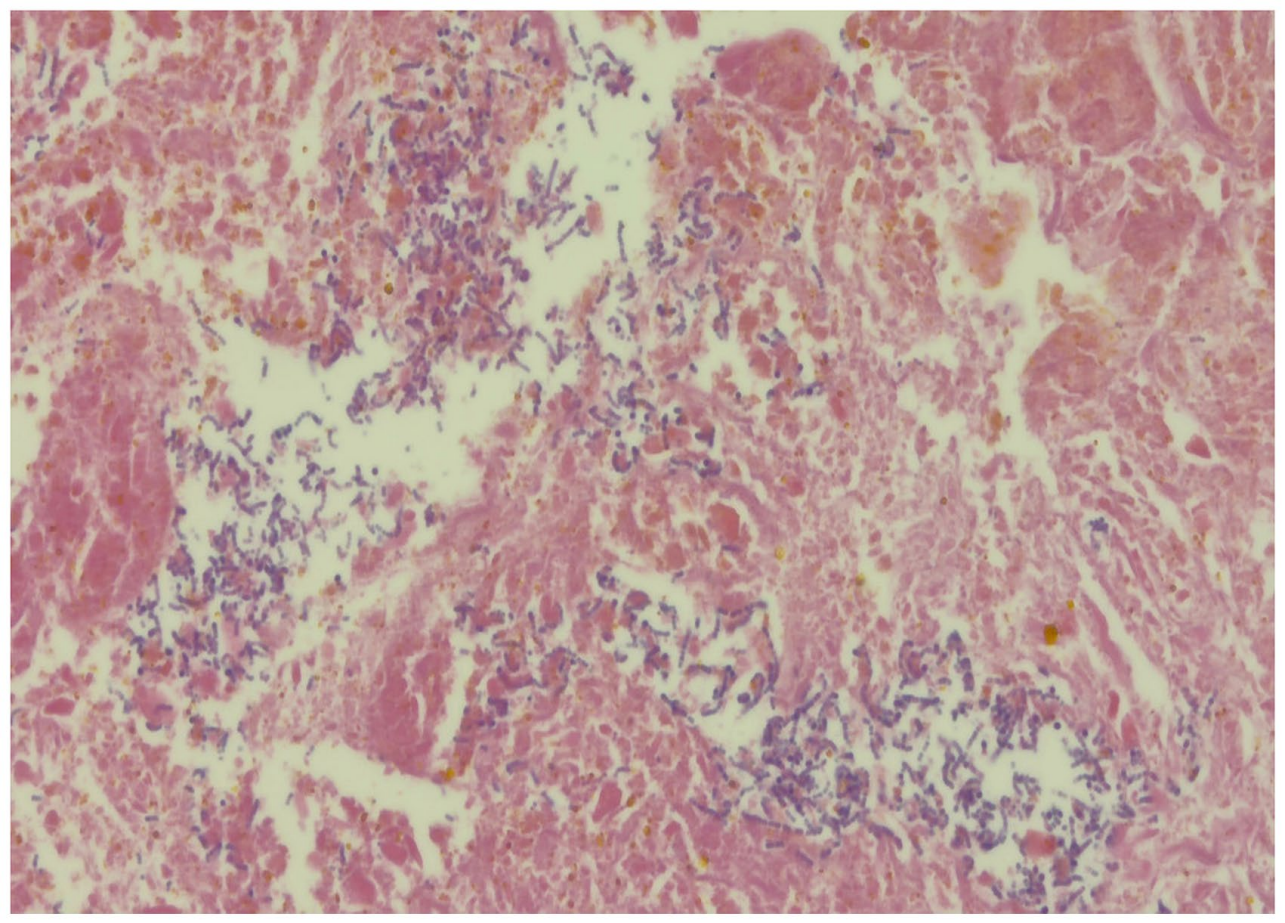
Pathological examination of the explanted liver.

**Figure 4:**
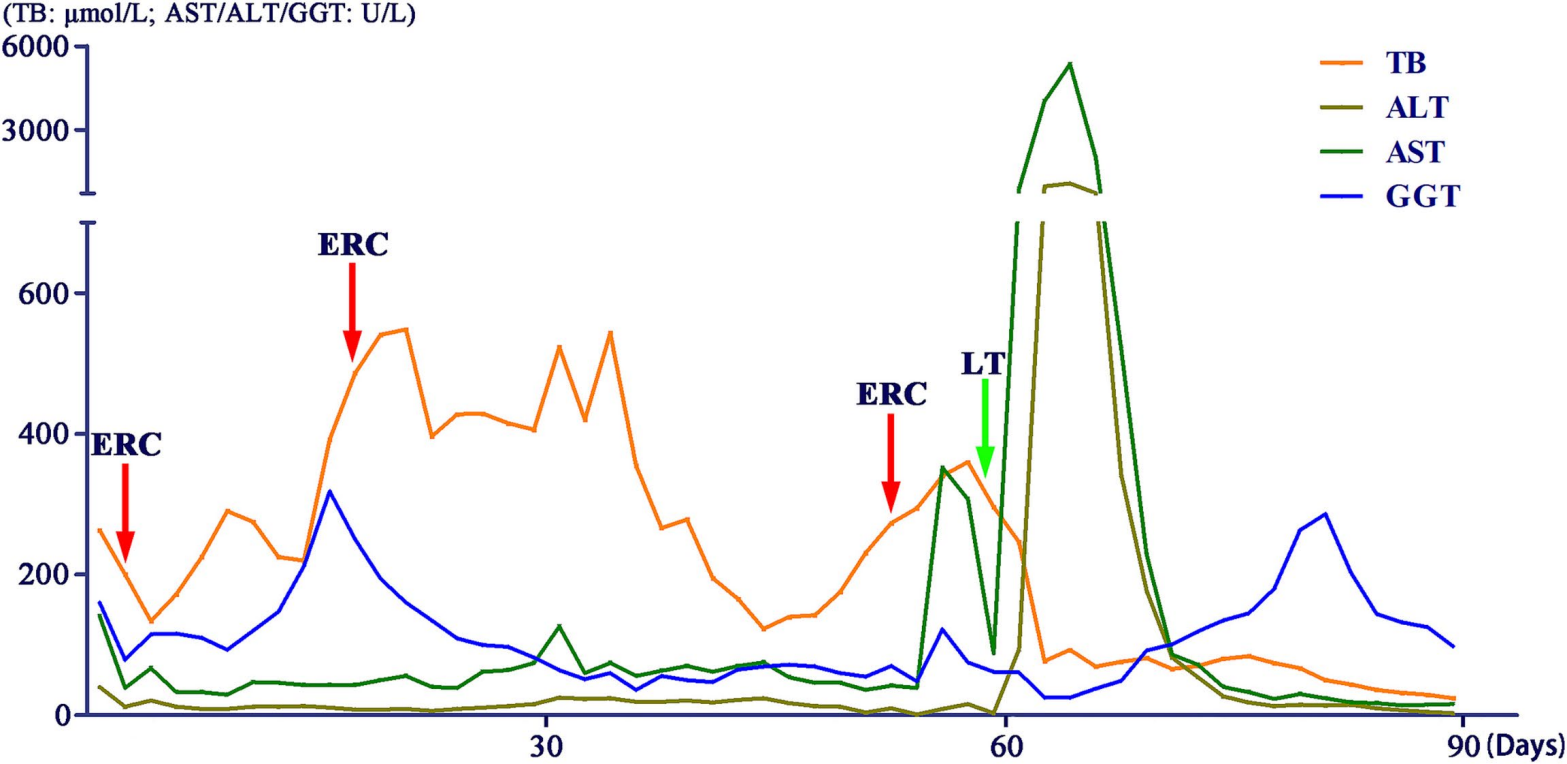
Changes in liver function indicators of the patient.

We conducted multiple endoscopic retrograde cholangiography (ERC) procedures, including balloon dilation, placement of biliary stents (Figure 1E), and nasobiliary drainage (Figure 1F) for the management of this patient. However, despite these interventions, the patient’s cholangitis and biliary obstruction continued to worsen. Ultimately, a second modified piggyback LT with bilioenteric anastomosis was conducted. During the dissection of the explanted liver, complete casts within the entire biliary tract were removed (Figure 2). Pathological examination revealed extensive cholestasis and infectious cholangitis with necrosis throughout the entire bile duct system (Figure 3). By post-transplantation day 20, the patient’s serum total bilirubin and transaminase levels had returned to normal values (Figure 4).

## Discussion

The incidence of NASs varies between 5 and 25% across different studies (2, 3). The occurrence of graft loss in NASs is frequently observed, with reported rates as high as 46% (6). BCS is a rare NAS post-liver transplant complication first described in 1975 (7). The incidence of BCS in LT patients ranges from approximately 2.1 to 3.6%, often necessitating re-transplantation (4). However, there is limited imaging evidence available regarding BCS, particularly complete hepatic cast formation, which plays a crucial role in comprehending the anatomy of the biliary tract and underlying pathological mechanisms.

This is a serious biliary complication that occurs following LT and is characterized by jaundice, increased cholestatic enzymes, and recurrent cholangitis. Imaging findings suggestive of a stricture at the biliary anastomotic site may lead to misdiagnosis as a biliary complication caused by AS. For AS, endoscopic interventions with ERC are recommended as first-line treatment (2, 8). Studies have consistently shown that complications associated with AS typically manifest during the early postoperative period, particularly within 3–6 months rather than later (9, 10). We concluded that this patient’s initial biliary obstruction and cholangitis symptoms were likely due to an NAS complication.

Etiologic mechanisms contributing to NASs include hepatic artery thrombosis or stenosis, ABO incompatibility, presence of T-tubes or bile leaks, ampullary dysfunction, chronic rejection, primary sclerosing cholangitis, circulatory instability, prolonged cold or warm ischemia, and marginal donor factors (2, 11, 12). This NAS case was due to ABO incompatibility. All bile ducts underwent immunological pathogenesis, resulting in complete cast formation, which is known as BCS. Buis CI et al. reported that NASs can lead to biliary destruction in four anatomical regions: hilar bifurcation, ducts between the first- and second-order branches, between second and third-order branches, and in the periphery of the liver (12). Involvement of ducts between second and third-order branches or in the periphery of the liver presents greater therapeutic challenges with a poorer prognosis, often necessitating re-transplantation for these patients (2, 13). Consequently, a second LT combined with bilioenteric anastomosis emerged as the sole viable treatment option for this patient.

This case report presents the most detailed replicas of the entire intrahepatic biliary tree documented to date, thereby enhancing our understanding of both the anatomical and pathological features of BCS.

## Data Availability

The raw data supporting the conclusions of this article will be made available by the authors, without undue reservation.
